# GLTSCR1 coordinates alternative splicing and transcription elongation of ZO1 to regulate colorectal cancer progression

**DOI:** 10.1093/jmcb/mjac009

**Published:** 2022-02-26

**Authors:** Fengyan Han, Beibei Yang, Mingyue Zhou, Qiong Huang, Minglang Mai, Zhaohui Huang, Maode Lai, Enping Xu, Honghe Zhang

**Affiliations:** Department of Pathology and Women's Hospital, Zhejiang University School of Medicine, Research Unit of Intelligence Classification of Tumor Pathology and Precision Therapy, Chinese Academy of Medical Sciences (2019RU042), Hangzhou 310058, China; Department of Pathology and Women's Hospital, Zhejiang University School of Medicine, Research Unit of Intelligence Classification of Tumor Pathology and Precision Therapy, Chinese Academy of Medical Sciences (2019RU042), Hangzhou 310058, China; Cancer Epigenetics Program, Wuxi School of Medicine, Jiangnan University, Wuxi 214122, China; Department of Pathology and Women's Hospital, Zhejiang University School of Medicine, Research Unit of Intelligence Classification of Tumor Pathology and Precision Therapy, Chinese Academy of Medical Sciences (2019RU042), Hangzhou 310058, China; Key Laboratory of Disease Proteomics of Zhejiang Province, Zhejiang University, Hangzhou 310058, China; Cancer Center, Zhejiang University, Hangzhou 310058, China; Department of Pathology and Women's Hospital, Zhejiang University School of Medicine, Research Unit of Intelligence Classification of Tumor Pathology and Precision Therapy, Chinese Academy of Medical Sciences (2019RU042), Hangzhou 310058, China; Cancer Epigenetics Program, Wuxi School of Medicine, Jiangnan University, Wuxi 214122, China; Key Laboratory of Disease Proteomics of Zhejiang Province, Zhejiang University, Hangzhou 310058, China; Cancer Center, Zhejiang University, Hangzhou 310058, China; Department of Pharmacology, China Pharmaceutical University, Nanjing 210009, China; Department of Pathology and Women's Hospital, Zhejiang University School of Medicine, Research Unit of Intelligence Classification of Tumor Pathology and Precision Therapy, Chinese Academy of Medical Sciences (2019RU042), Hangzhou 310058, China; Key Laboratory of Disease Proteomics of Zhejiang Province, Zhejiang University, Hangzhou 310058, China; Cancer Center, Zhejiang University, Hangzhou 310058, China; Department of Pathology and Women's Hospital, Zhejiang University School of Medicine, Research Unit of Intelligence Classification of Tumor Pathology and Precision Therapy, Chinese Academy of Medical Sciences (2019RU042), Hangzhou 310058, China; Key Laboratory of Disease Proteomics of Zhejiang Province, Zhejiang University, Hangzhou 310058, China; Cancer Center, Zhejiang University, Hangzhou 310058, China

**Keywords:** colorectal cancer, alternative splicing, transcription elongation, GLTSCR1

## Abstract

Alternative splicing (AS) and transcription elongation are vital biological processes, and their dysregulation causes multiple diseases, including tumors. However, the coregulatory mechanism of AS and transcription elongation in tumors remains unclear. This study demonstrates a novel AS pattern of tight junction protein 1 (ZO1) regulated by the RNA polymerase II elongation rate in colorectal cancer (CRC). Glioma tumor suppressor candidate region gene 1 (GLTSCR1) decreases the transcription elongation rate of ZO1 to provide a time window for binding of the splicing factor HuR to the specific motif in intron 22 of ZO1 and spliceosome recognition of the weak 3′ and 5′ splice sites in exon 23 to promote exon 23 inclusion. Since exon 23 inclusion in ZO1 suppresses migration and invasion of CRC cells, our findings suggest a novel potential therapeutic target for CRC.

## Introduction

Alternative splicing (AS) is a key biological event that increases the coding efficiency of eukaryotic genes and increases protein diversity (Baralle and Giudice, 2017; Gallego-Paez et al., 2017). The systematic and dynamic regulation of AS is indispensable for sustaining the lives of organisms. Based on the splice site and the interaction location, the regulatory elements of AS are classified as *cis*-regulatory sequences and *trans*-acting factors. *Cis*-regulatory sequences include intronic or exonic splicing enhancers and silencers, while *trans*-acting factors bind to *cis*-regulatory sequences to regulate AS (Naftelberg et al., 2015). To date, some evidence indicates that transcription and AS are not independent events but instead can occur concurrently. AS might even be coupled to the transcription machinery (Kornblihtt et al., 2013); thus, the process of AS is thought to be more complex than previously expected. In addition to splicing factors and interactive regulatory elements, many other factors, including chromatin remodeling, histone modification, nucleosome positioning, and the RNA polymerase II (Pol II) transcription elongation rate, also affect AS (Luco et al., 2010; Tauber et al., 2020). Two different models have been reported to explain the mechanism by which Pol II participates in the pre-mRNA AS process: in one model, termed the recruitment model, Pol II enriches abundant splicing factors in the vicinity of the pre-mRNA (Das et al., 2007; Huang et al., 2012); in the other model, defined as the kinetic model, the Pol II elongation rate dynamically regulates AS events (de la Mata et al., 2003). Although the regulation of AS by Pol II has been proposed and clarified along with the development of next-generation sequencing, the detailed molecular mechanism remains unclear, and the roles of cotranscriptional splicing in tumorigenesis need to be elucidated.

Abundant aberrant AS events have been discovered in multiple types of cancer and contribute to tumor development. These splicing variants might be considered a reservoir of new cancer-specific markers and neoantigens (Kahles et al., 2018; Frankiw et al., 2019). Tight junction protein 1 (ZO1) is a well-known cytoplasmic scaffolding and tight junction protein (Fanning et al., 1998), and the ZO1 exon 23 (ZO1 E23) AS event has been well documented in many studies. Our previous study showed that ZO1 E23 AS is a pivotal AS event in colorectal cancer (CRC) progression and is regulated by the prototypical serine–arginine (SR) protein SR-rich splicing factor 6 (SRSF6) (Wan et al., 2019). In addition to SRSF6, the RNA-binding proteins (RBPs) heterogeneous nuclear ribonucleoprotein L (hnRNPL) and RNA-binding motif protein 47 (RBM47) also affect ZO1 E23 AS (Heiner et al., 2010; Kim et al., 2019). However, whether the ZO1 AS event is coupled to transcriptional regulation has not been reported to date.

Glioma tumor suppressor candidate region gene 1 (GLTSCR1) is located on chromosome 19q13.33 and exhibits frequent allelic loss in human diffuse gliomas (Smith et al., 2000; McKean-Cowdin et al., 2009). GLTSCR1 regulates gene expression and genome integrity by mediating the formation of the mammalian switching defective/sucrose non-fermenting chromatin remodeling complex (Alpsoy and Dykhuizen, 2018). Our previous study showed that GLTSCR1 interacts with BRD4 to regulate gene transcription elongation, which reduces the capability for CRC metastasis (Han et al., 2019). However, it is still poorly understood whether GLTSCR1 regulates AS by controlling transcription elongation. In the present study, we demonstrated that GLTSCR1 decreased the ZO1 transcription elongation rate to provide a longer time window for the splicing factor HuR to recognize the 3′ and 5′ weak splice sites in ZO1 E23, which resulted in E23 inclusion in ZO1 and suppressed CRC progression.

## Results

### GLTSCR1 regulates ZO1 E23 AS

**Figure 1:**
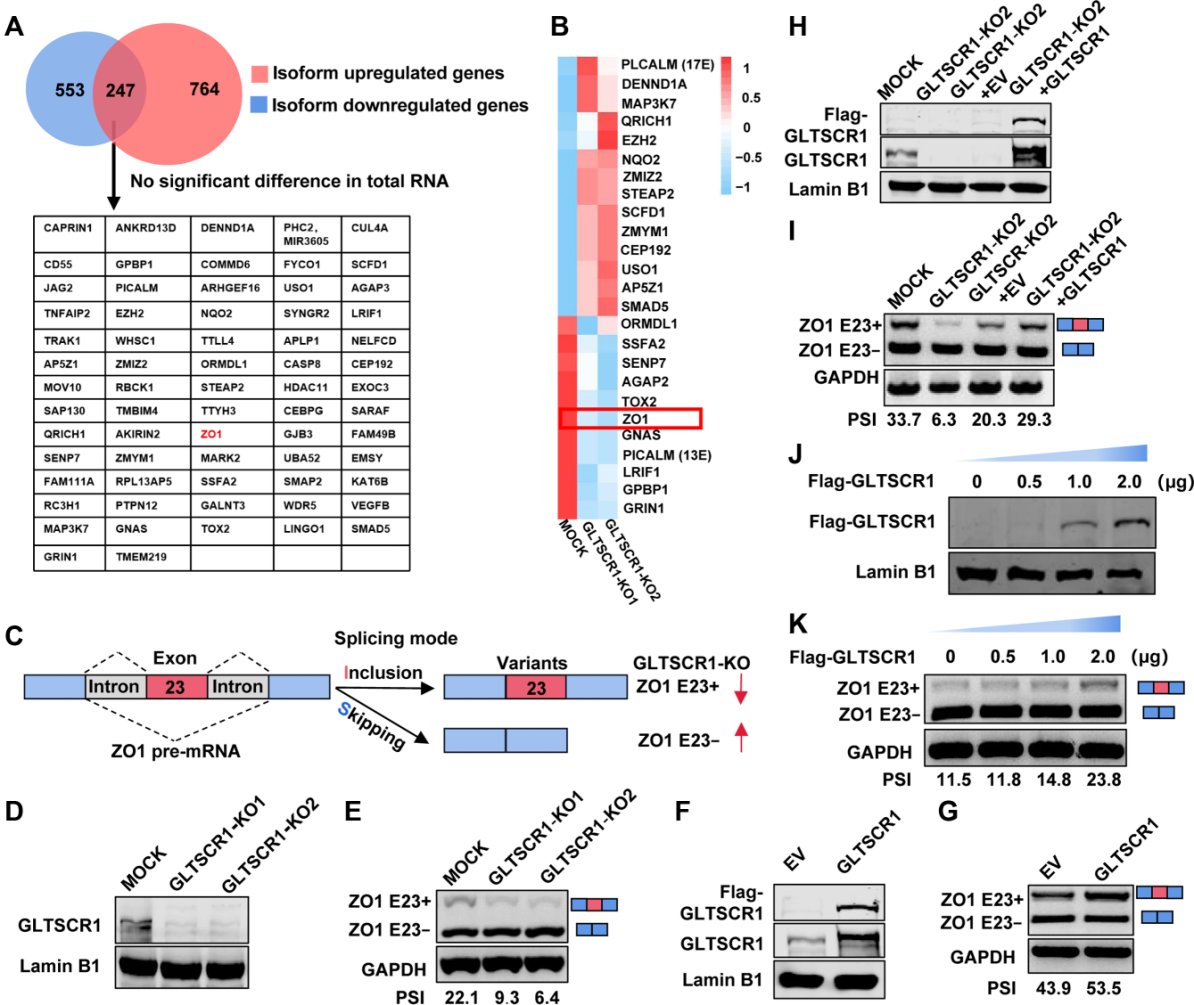
GLTSCR1 regulates ZO1 E23 AS. (**A**) Venn diagram showing that 67 genes had opposite change trends for different isoforms but no significant change in the total mRNA expression level when GLTSCR1 was knocked out in HCT116 cells. (**B**) Heat map showing the expression of exon inclusion isoforms in different exon skipping events in the indicated genes. (**C**) Schematic diagram of ZO1 E23 AS. (**D**) Western blotting was performed to confirm the GLTSCR1 KO efficiency. (**E**) The expression of two ZO1 isoforms in HCT116 MOCK, GLTSCR1-KO1, and GLTSCR1-KO2 cells was verified by RT–qPCR. PSI index is shown. (**F** and **G**) HCT116 cells were transfected with control empty vector (EV) and Flag-tagged GLTSCR1 expression constructs. (**F**) Exogenous and endogenous expression was confirmed by western blotting using an anti-FLAG antibody and an anti-GLTSCR1 antibody, respectively. Lamin B1 was used as the loading control. (**G**) RT–qPCR analysis of ZO1 E23+/E23− isoform expression. (**H** and **I**) GLTSCR1-KO2 cells were transfected to overexpress Flag-tagged GLTSCR1 or control EV. (**H**) Exogenous and endogenous expression was confirmed by western blotting using an anti-Flag antibody and an anti-GLTSCR1 antibody, respectively. (**I**) RT–qPCR analysis of ZO1 E23+/E23− isoform expression. (**J** and **K**) Increasing doses of Flag-GLTSCR1 were transfected into GLTSCR1-KO2 cells. (**J**) The expression of Flag-GLTSCR1 was evaluated by western blotting using an anti-Flag antibody. (**K**) The ZO1 splicing pattern was examined by RT–qPCR.

To discover AS events regulated by GLTSCR1, we reanalyzed our previous RNA sequencing (RNA-seq) data (PRJNA517374) from GLTSCR1-knockout (KO) CRC cells. GLTSCR1 KO resulted in 1011 genes with upregulated isoforms and 800 genes with downregulated isoforms. Among these genes, 247 had two different isoforms that displayed opposite expression patterns. Then, 67 genes with no significant change in the total mRNA level were selected for further analysis (Figure 1A). Moreover, we identified 25 exons skipping AS events, including both exon inclusion and exclusion splicing events, regulated by GLTSCR1 (Figure 1B; Supplementary Figure S1A and B). Interestingly, our previous study demonstrated that ZO1 E23 AS was regulated by SRSF6; however, GLTSCR1 also affected the ZO1 E23 AS event (Figure 1C). In HCT116 GLTSCR1-KO cells (Figure 1D), we further confirmed that the expression of the ZO1 E23 inclusion splice variant (E23+) was significantly decreased by GLTSCR1 KO (Figure 1E). When we overexpressed GLTSCR1 in HCT116 cells (Figure 1F), ZO1 E23+ expression was significantly increased (Figure 1G). Consistent with this finding, when we re-expressed GLTSCR1 in HCT116 GLTSCR1-KO cells (Figure 1H), ZO1 E23+ expression was rescued (Figure 1I). As GLTSCR1 expression increased (Figure 1J), ZO1 E23+ expression gradually increased (Figure 1K). Collectively, these data indicated that ZO1 E23 splicing was one of the AS events regulated by GLTSCR1.

### GLTSCR1 regulates the inclusion of ZO1 E23 and inhibits migration and invasion of CRC cells

**Figure 2:**
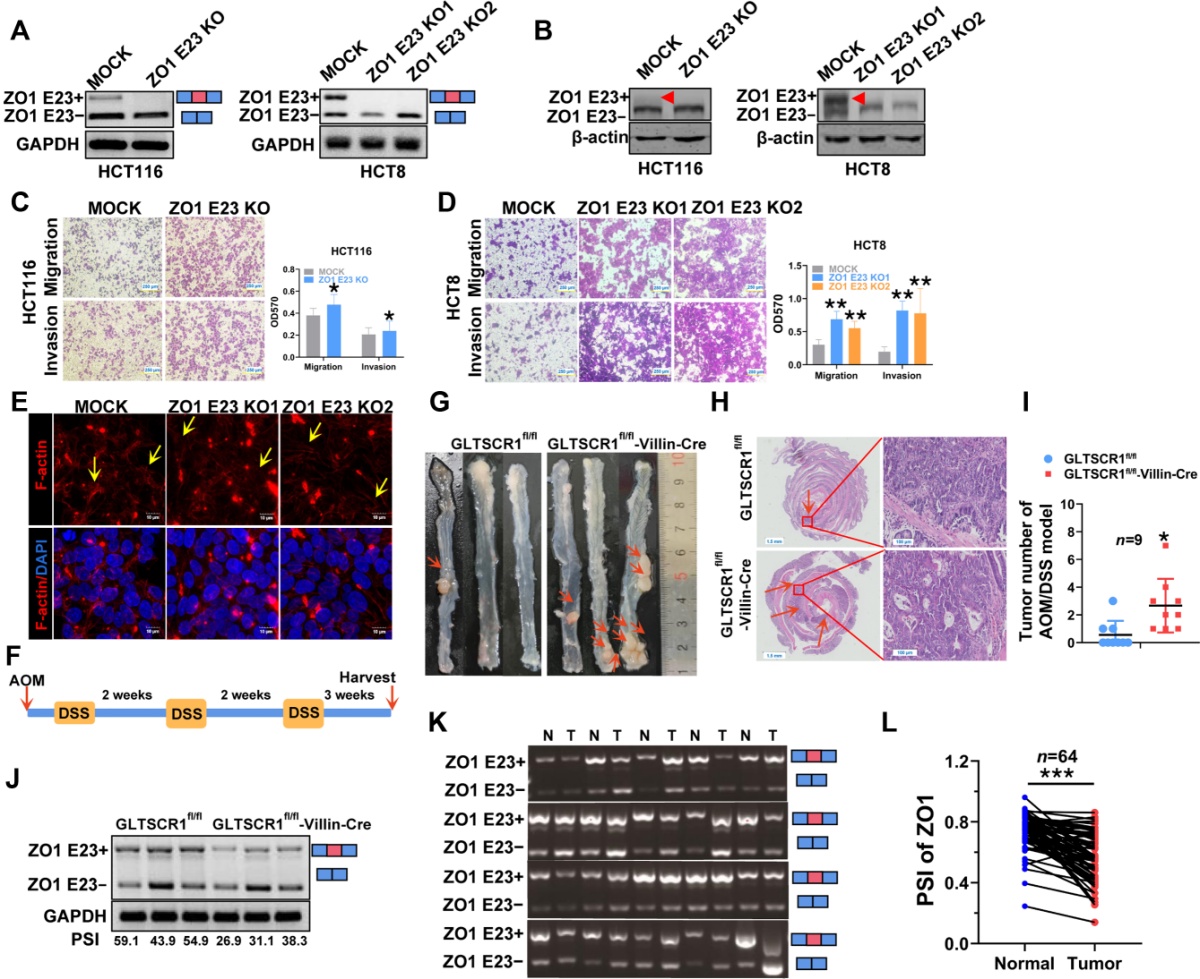
GLTSCR1 regulates the inclusion of ZO1 E23 and inhibits migration and invasion of CRC cells. (**A** and **B**) RT–qPCR and western blotting were performed to detect ZO1 E23 splice isoform expression in ZO1 E23 KO HCT116 and HCT8 cells. The arrow shows the protein expression of ZO1 E23+. (**C** and **D**) Transwell assay was performed to investigate changes in migration and invasion induced by ZO1 E23 KO in HCT116 (**C**) and HCT8 (**D**) cells; the histogram on the right shows the quantitative analysis results. Scale bar, 250 µm. The data are presented as mean ± SD; statistical significance was assessed by unpaired *t*-test. *P < 0.05, **P < 0.01, ***P < 0.001; *n* = 3. (**E**) Immunofluorescence staining showing the assembly of actin stress fibers in ZO1 E23 KO HCT8 cells. Cells were immunostained with phalloidin (red) and DAPI (blue). Scale bar, 10 µm. (**F**) Schematic overview of the inflammation-induced colitis-associated CRC model. (**G**) Representative images of tumors in the distal colon and rectum of control and GLTSCR1^fl/fl^-Villin-Cre mice; the arrows indicate the tumor foci. (**H**) H&E staining of tumor foci from **G**; the arrows indicate the tumor foci. Scale bar, 1.5 mm (left) and 100 µm (right). (**I**) Quantification of intestinal lesion numbers in control and GLTSCR1^fl/fl^-Villin-Cre mice. Statistical significance was assessed by unpaired *t*-test. *P < 0.05, **P < 0.01, ***P < 0.001; *n* = 9. (**J**) RT–qPCR analysis of ZO1 E23 splice isoform expression in control and GLTSCR1^fl/fl^-Villin-Cre mouse tumor foci. (**K**) RT–qPCR analysis of ZO1 E23 splice isoform expression in clinical CRC and matched normal tissues. (**L**) PSI index of ZO1 E23 in clinical CRC and matched normal tissues. Statistical significance was assessed by paired *t*-test. *P < 0.05, **P < 0.01, ***P < 0.001; *n* = 64.

As a major component of the tight junction complex, ZO1 prevents cell migration and tumor metastasis in breast cancer (Thion et al., 2015), pancreatic cancer (Liu et al., 2018), and other cancers. Recently, researchers reported that the circular RNA isoleucyl-tRNA synthetase (IARS) increased endothelial monolayer permeability and promoted tumor invasion and metastasis by downregulating ZO1 (Li et al., 2018). In addition to the circular RNA IARS, microRNAs can also regulate the expression of ZO1 (Zhou et al., 2014; Zeng et al., 2018). Our previous study reported that specifically knocking down the ZO1 E23+ variant in HCT8 cells with individual small interfering RNAs (siRNAs) caused dramatic increases in migration and invasion but knocking down the ZO1 E23− variant in RKO and SW620 cells had no biological effect (Wan et al., 2019). To further verify the roles of ZO1 splicing variants in CRC, we deleted E23 in HCT116 and HCT8 cells, which have high ZO1 E23+ expression, through CRISPR/Cas9 gene editing (Supplementary Figure S2A). ZO1 E23 was verified to be successfully knocked out at both mRNA level (Figure 2A) and protein level (Figure 2B), and the potential for both migration and invasion was found to be significantly increased in HCT116 (Figure 2C) and HCT8 cells (Figure 2D). As a cell adhesion molecule, ZO1 might not only restrict cell movement but also maintain the cytoskeleton. To determine whether ZO1 E23 is essential for cell adhesion, we used an immunofluorescence assay to detect the localization of the ZO1 E23− variant. The ZO1 E23− variant was colocalized with E-cadherin in cells, similar to the ZO1 E23+ variant (Supplementary Figure S2B). Then, we stained F-actin to investigate whether ZO1 E23 AS affects the cytoskeleton. As expected, deletion of ZO1 E23 decreased F-actin enrichment (Figure 2E), indicating that ZO1 E23 exclusion resulted in a defect in the F-actin distribution and promoted CRC cell migration and invasion.

To further identify the biological function of GLTSCR1 in regulating ZO1 E23 AS *in vivo*, we established a mouse model with conditional deletion of GLTSCR1 in intestinal epithelial cells (GLTSCRΔIEC) by breeding GLTSCR1^fl/fl^ mice with Villin-Cre mice (GLTSCR1^fl/fl^-Villin-Cre), which start to express Cre recombinase on approximately embryonic day 10 (el Marjou et al., 2004; Supplementary Figure S2C). Through genotyping (Supplementary Figure S2D and E), we confirmed that the specific GLTSCR1 KO mouse model was successfully established. Furthermore, we used azoxymethane (AOM) and dextran sodium sulfate (DSS) to establish a mouse model of colitis-associated CRC to mimic the progression of CRC (Figure 2F). As shown in Figure 2G, compared with GLTSCR1^fl/fl^ mice, GLTSCR1^fl/fl^-Villin-Cre mice exhibited a significantly increased number of tumors in the colorectum. Hematoxylin and eosin (H&E) staining showed that more and larger tumors formed in the colorectum when GLTSCR1 was conditionally deleted in intestinal epithelial cells (Figure 2H and I). The ZO1 E23+ variant was significantly downregulated in the tumors of GLTSCR1^fl/fl^-Villin-Cre mice compared with that of GLTSCR1^fl/fl^ mice (Figure 2J), consistent with the results observed *in vitro*. Then, we assessed ZO1 E23 AS in 64 paired CRC samples by reverse transcription–quantitative polymerase chain reaction (RT–qPCR). ZO1 E23+ variant expression was significantly reduced and E23− variant expression was increased in tumor samples (Figure 2K). The % spliced in (PSI) index of ZO1 E23 in CRC tissues was much lower than that in the matched normal tissues (Figure 2L). Taken together, these findings indicated that the ZO1 E23 AS event generated two different transcriptional variants and that the ZO1 E23+ variant showed a suppression in migration and invasion *in vitro*. Moreover, GLTSCR1 deletion promoted ZO1 E23 exclusion, which facilitated the development of CRC *in vivo*.

### GLTSCR1 regulates ZO1 E23 AS by inhibiting transcription elongation

**Figure 3:**
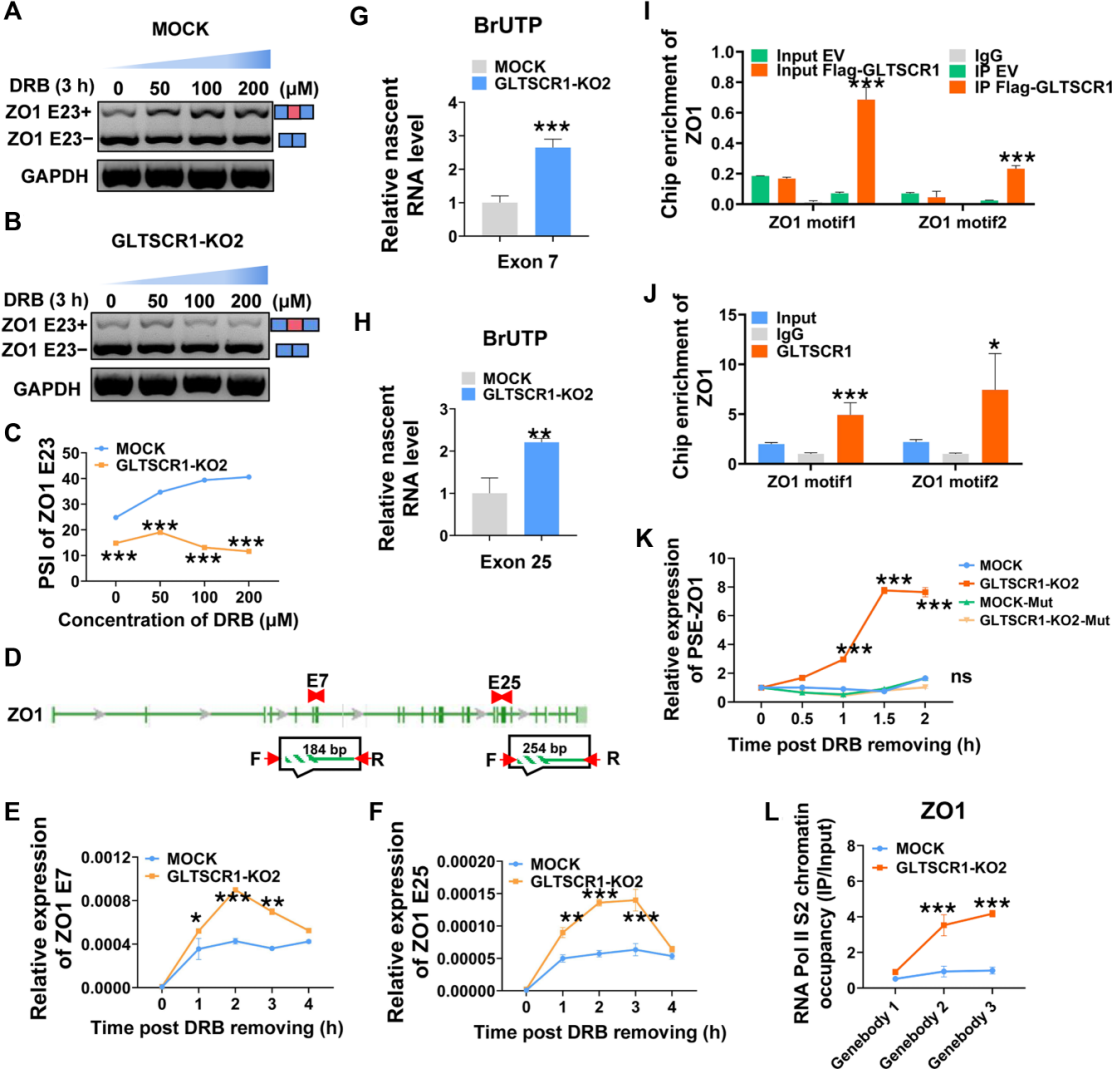
GLTSCR1 regulates ZO1 E23 AS by inhibiting transcription elongation. (**A**−**C**) RT–qPCR analysis of ZO1 E23 splicing isoform expression after treatment with DRB (0, 50, 100, and 200 µM) for 3 h to inhibit RNA synthesis in HCT116 MOCK (**A**) and GLTSCR1-KO2 (**B**) cells and the PSI index of ZO1 E23 (**C**). (**D**) Schematic overview of the ZO1 pre-mRNA detection strategy. (**E** and **F**) Transcription efficiency of ZO1 in HCT116 MOCK and GLTSCR1-KO2 cells, as determined by RT–qPCR using the ZO1 E7 primer (**E**) and ZO1 E25 primer (**F**). (**G** and **H**) Detection of nascent ZO1 RNA in HCT116 MOCK and GLTSCR1-KO2 cells through RNA pulldown with an anti-BrdU antibody using the ZO1 exon 7 primer (**G**) and ZO1 exon 25 primer (**H**). (**I** and **J**) ChIP–PCR was performed with an anti-Flag antibody (**I**) and an anti-GLTSCR1 antibody (**J**) to detect the DNA-binding capacity of Flag-GLTSCR1 and endogenous GLTSCR1, respectively, to the ZO1 gene. (**K**) Exogenous transcription efficiency of ZO1 in HCT116 MOCK and GLTSCR1-KO2 cells transfected with two ZO1 promoter minigenes with mutated GLTSCR1 DNA-binding sites and treated with DRB to inhibit RNA synthesis, as determined by RT–qPCR. (**L**) ChIP–PCR was performed with an anti-RNA Pol II S2 antibody to detect RNA Pol II chromatin occupancy at multiple sites in the ZO1 gene in MOCK and GLTSCR1-KO2 cells. The data are presented as mean ± SD; statistical significance was assessed by unpaired *t*-test. *P < 0.05, **P < 0.01, ***P < 0.001; *n* = 3.

The mechanisms of AS regulation by both *cis*-regulatory sequences and *trans*-acting factors have been extensively studied (Chasin, 2007; Martinez-Contreras et al., 2007). However, recent studies have highlighted the roles of transcriptional regulatory factors, including chromatin remodeling, histone modification, and the Pol II elongation rate, in AS. Our previous study demonstrated that GLTSCR1 inhibited CRC metastasis by inhibiting transcription elongation. To investigate whether ZO1 E23 AS is coupled with transcription elongation regulated by GLTSCR1, we treated GLTSCR1-KO and MOCK cells with different concentrations of 5,6-dichlorobenzimidazole 1-β-D-ribofuranoside (DRB), an inhibitor of RNA synthesis that causes premature transcription termination (Chodosh et al., 1989). ZO1 E23 inclusion was enhanced with increasing DRB concentration in MOCK cells (Figure 3A) but not in GLTSCR-KO cells (Figure 3B). In addition, the PSI index was significantly lower in GLTSCR-KO cells than in MOCK cells (Figure 3C). Furthermore, we assessed the effects of GLTSCR1 on the transcription elongation rate of ZO1 by designing primers specific for exon 7 (E7) and exon 25 (E25) of ZO1 (Figure 3D). GLTSCR1 KO significantly increased the transcription elongation rate of ZO1 (Figure 3E and F). Although the RNA-seq data (Figure 1B) showed that GLTSCR1 also affected the phosphatidylinositol binding clathrin assembly protein (PICALM) exon 13 AS event, the transcription elongation rate of PICALM was not changed by GLTSCR1 depletion (Supplementary Figure S3A). These data showed a specific effect of GLTSCR1 on coupling ZO1 AS with transcription elongation. Additionally, we used an anti-5-bromo-2′-deoxyuridine (BrdU) antibody to pull down BrdU-labelled nascent ZO1 RNA and found more nascent ZO1 RNA synthesized in GLTSCR1-KO cells than in MOCK cells (Figure 3G and H).

Based on the GLTSCR1-binding motif identified in our previous study (Han et al., 2019), we found that two GLTSCR1-binding motifs were located near the transcription start site (TSS) of ZO1 (Supplementary Figure S3B). Exogenous and endogenous chromatin immunoprecipitation–PCR (ChIP–PCR) assays showed that both binding motifs in ZO1 were enriched by GLTSCR1 (Figure 3I and J). Furthermore, to investigate whether these two binding motifs are important for controlling the ZO1 transcription elongation rate, we mutated these two GLTSCR1 DNA-binding sites in the ZO1 promoter and used DRB to inhibit RNA synthesis. As shown in Figure 3K, GLTSCR1 KO significantly increased the transcription elongation rate of ZO1 in the minigene model, consistent with the findings for endogenous transcription elongation. Moreover, ZO1 mutation abolished the effects of GLTSCR1 on the transcription elongation rate of ZO1. Collectively, these results indicated that GLTSCR1 reduced the transcription elongation rate of ZO1 by specifically binding to two binding motifs upstream of the ZO1 TSS. To further confirm the regulatory role of GLTSCR1 in ZO1 transcription elongation, we monitored Pol II release and slippage on the ZO1 gene body after DRB-induced release. When GLTSCR1 was knocked out, the gene body of ZO1 exhibited increased Pol II chromatin occupancy, as determined by ChIP–PCR (Figure 3L; Supplementary Figure S3C). Taken together, these data suggested that GLTSCR1 specifically reduced the transcription elongation rate of ZO1, which caused an increase in ZO1 E23 inclusion.

### The weak 3′ and 5′ splice sites in ZO1 E23 cooperate with transcription elongation to regulate AS

**Figure 4:**
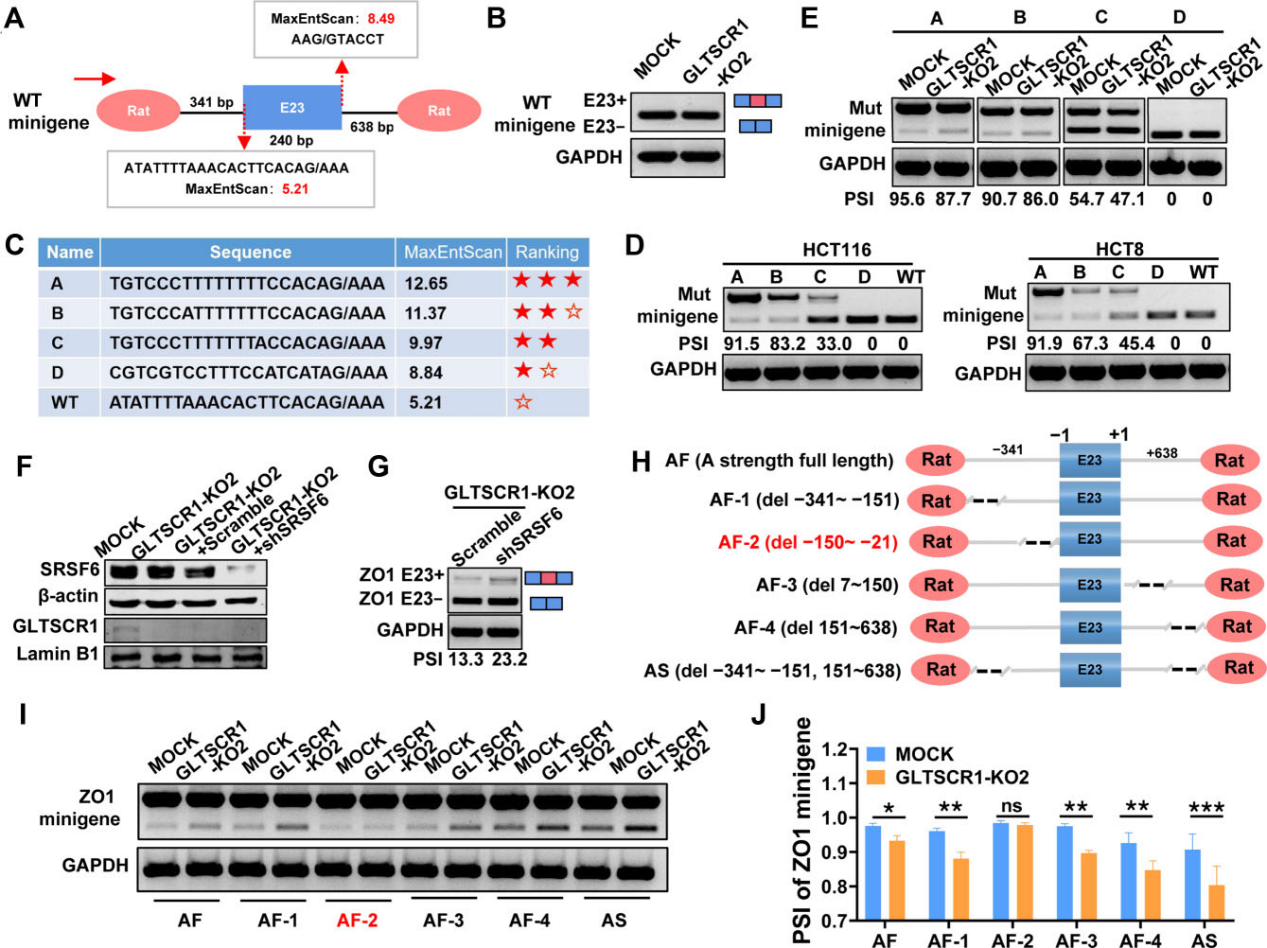
The weak 3′ and 5′ splice sites in ZO1 E23 cooperate with transcription elongation to regulate AS. (**A**) Diagram of the ZO1 minigene construct, which was designed to contain the genomic sequence of the ZO1 gene, including E23, the flanking upstream and downstream introns of E23, and the constitutive rat exon. The splice site scores of the ZO1 E23 3′ and 5′ splice sites were obtained using MaxEnt scores. (**B**) The wild-type (WT) ZO1 minigene was transfected into HCT116 MOCK and GLTSCR1-KO2 cells, and RT–qPCR was then performed. (**C**) Schematic diagram of the mutant (Mut) ZO1 minigenes. The 3′ splice site in the internal exon was replaced by exchanging a fragment to generate four different 3′ spice sites of variable strengths. (**D**) Four Mut ZO1 minigenes with different 3′ splice site strengths and WT ZO1 minigene were transfected separately into HCT116 and HCT8 cells, and RT–qPCR was then performed. (**E**) Four Mut ZO1 minigenes with different 3′ splice site strengths were transfected into HCT116 MOCK and GLTSCR1-KO2 cells, and RT–qPCR was then performed. (**F**) Western blotting analysis of SRSF6 protein level in scramble and shSRSF6 GLTSCR1-KO2 cells. (**G**) RT–qPCR analysis of ZO1 E23 splice isoform expression in scramble and shSRSF6 GLTSCR1-KO2 cells. (**H**) Diagram of ZO1 minigene constructs with deletion of intronic regions. The positions of the deleted nucleotides in the minigene constructs are shown in brackets. (**I**) The deletion minigene constructs were transfected into HCT116 MOCK and GLTSCR1-KO2 cells, and RT–qPCR was then performed. (**J**) Histogram showing the quantitative analysis results for minigenes with different PSI indexes in HCT116 MOCK and GLTSCR1-KO2 cells. The data are presented as mean ± SD; statistical significance was assessed by unpaired *t*-test. *P < 0.05, **P < 0.01, ***P < 0.001; ns, not statistically significant; *n* = 3.

Decreasing the Pol II elongation rate could provide a time window for Pol II-associated splicing factors to recognize weak splice sites, leading to preferential exon inclusion (de la Mata et al., 2003). Therefore, it is a requirement for GLTSCR1 to regulate ZO1 E23 inclusion by slowing ZO1 transcription elongation, because ZO1 E23 contains a weak splice site. First, we used maximum entropy (MaxEnt) scores to predict the splice site strength of ZO1 E23 (Tsai and Wang, 2012). As expected, the 3′ splice site score was 5.21, and the 5′ splice site score was 8.49, as both were thought to be weak splice sites (Figure 4A). Then, we used the pSpliceExpress (pSE) minigene plasmid to construct a wild-type ZO1 E23 minigene containing the full length of both intron 22 and intron 23. Because a GLTSCR1-binding motif is located upstream of the restriction enzyme site ∼202 base pairs from the TSS, and ChIP–PCR assay showed that the minigene promoter was enriched by GLTSCR1 (Supplementary Figure S4A), this approach provided an option for us to investigate the regulatory effect of GLTSCR1 on ZO1 E23 AS. As shown in Figure 4B, no E23 inclusion was observed from the wild-type ZO1 E23 minigene. The wild-type ZO1 E23 minigene retains the weak splice site, whereas the pSE minigene contains two constitutively expressed rat insulin exons (Figure 4A) that are spliced together in most cases, which served as a positive control. With the Alternative Splicing Database (ASD) (Thanaraj et al., 2004), we constructed a series of mutant ZO1 E23 minigenes with mutation of the 3′ splice site to generate different splice site strengths (Figure 4C). Consistently, when we transfected these mutant minigenes into HCT116 and HCT8 CRC cells, the inclusion percentage of ZO1 E23 was increased as the splice site strength increased (Figure 4D). This pattern suggested that the weak splice site of ZO1 E23 was required for E23 skipping. To evaluate the impact of GLTSCR1 and the elongation rate on ZO1 E23 AS, we also transfected these mutant minigenes into GLTSCR1-KO and MOCK cells to determine whether the impact of GLTSCR1 on E23 AS was abolished by these strong splice sites. Interestingly, even when the splice site score was 12.65, indicating a high enough strength for inclusion events to neglect the regulatory effect of the elongation rate, GLTSCR1 KO still promoted ZO1 E23 exclusion (Figure 4E). This result implied that another splicing factor might participate in ZO1 E23 AS when GLTSCR1 is knocked out. In our previous study, the splicing factor SRSF6 was found to facilitate ZO1 E23 exclusion by directly binding to the motif in E23 (Wan et al., 2019). Therefore, we knocked down SRSF6 in GLTSCR1-KO cells (Figure 4F) and then assessed ZO1 E23 AS. As shown in Figure 4G, SRSF6 knockdown still increased ZO1 E23 inclusion in GLTSCR1-KO cells, demonstrating that GLTSCR1 and SRSF6 are two independent regulators of ZO1 E23 AS.

To further screen coregulatory splicing factors cooperating with GLTSCR1 for ZO1 E23 AS, we needed to determine the key motif that might bind to a splicing factor. Therefore, we generated different truncated minigenes of ZO1 E23 with A-level (Figure 4C) strength at the 3′ and 5′ splice sites (Figure 4H). After transfection of these truncated minigenes into GLTSCR1-KO and MOCK cells, only the truncated minigene with deletion of the −150 to −21 motif at the 3′ splice site of E23 rescued ZO1 E23 AS regulated by GLTSCR1 KO (Figure 4I and J). Collectively, these results indicated that GLTSCR1 decreased the transcription elongation rate of ZO1 to facilitate the recognition of the −150 to −21 motif by Pol II-associated splicing factors to promote ZO1 E23 inclusion.

### HuR binds to intron 22 of ZO1 and promotes ZO1 E23 inclusion

**Figure 5:**
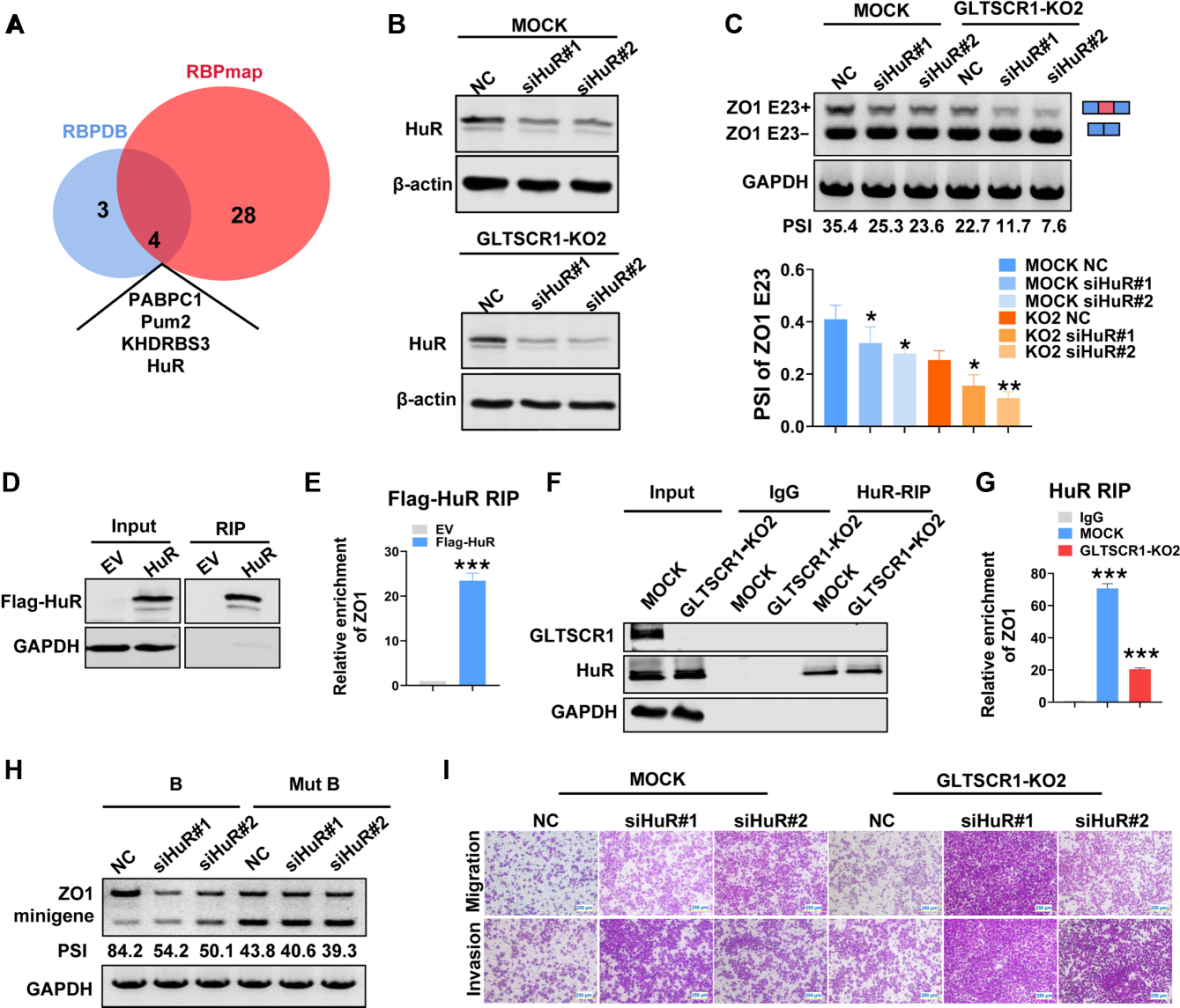
HuR binds to intron 22 of ZO1 and promotes ZO1 E23 inclusion. (**A**) Venn diagram of proteins binding to the ZO1 E23 upstream intronic region identified by RBPDB and RBPmap. (**B** and **C**) HuR was knocked down by siRNA in HCT116 MOCK and GLTSCR1-KO2 cells. (**B**) Western blotting was performed to detect HuR expression. (**C**) RT–qPCR was performed to detect ZO1 E23 splice isoform expression. The histogram shows quantitative analysis results for the PSI index. (**D**) Proteins immunoprecipitated in the exogenous RIP assay were verified by western blotting using an anti-FLAG antibody. (**E**) RT–qPCR results showing ZO1 pre-mRNA binding to Flag-tagged HuR in HEK293T cells via RIP. (**F**) Proteins immunoprecipitated in the endogenous RIP assay were verified by western blotting using an anti-GLTSCR1 antibody. (**G**) RT–qPCR results showing ZO1 pre-mRNA binding to HuR in HCT116 MOCK and GLTSCR-KO2 cells via RIP. (**H**) RT–qPCR was performed to detect ZO1 E23 splice isoform expression in cells transfected with the B-strength splice site minigene (B) or HuR-binding site-deleted mutant minigene (Mut B) and with or without HuR knockdown by siRNA. (**I**) Transwell assay to investigate changes in migration and invasion induced by HuR knockdown in HCT116 MOCK and GLTSCR1-KO2 cells. Scale bar, 250 µm. The data are presented as mean ± SD; statistical significance was assessed by unpaired *t*-test. *P < 0.05, **P < 0.01, ***P < 0.001; ns, not statistically significant; *n* = 3.

To discover the coregulatory splicing factor cooperating with GLTSCR1 for ZO1 E23 AS, we used the RNA Binding Proteins DataBase (RBPDB) and RBPmap (a tool for mapping RBPs), two web servers for mapping binding sites of RBPs (Cook et al., 2011; Paz et al., 2014), to scan the −150 to −21 sequences in E23 and predict RBP binding sites. A total of 7 and 32 RBPs were predicted to bind to the −150 to −21 motif in ZO1 E23 by RBPDB and RBPmap, respectively, and only 4 proteins were predicted by both servers (Figure 5A). Then, 14 splicing-associated RBPs were selected for detection of their mRNA expression in GLTSCR1-KO and MOCK cells (Supplementary Figure S5A and B). Based on these expression levels, we finally selected eight candidate genes for further investigation. We used siRNA to knock down these eight candidate genes in GLTSCR1-KO and MOCK cells (Supplementary Figure S5C). When hnRNPL and SRSF1 were knocked down, ZO1 E23 inclusion was increased in both GLTSCR1-KO and MOCK cells (Supplementary Figure S5D–F). However, only HuR knockdown (Figure 5B) decreased ZO1 E23 inclusion, with a lower level in GLTSCR1-KO cells than in MOCK cells (Figure 5C). Furthermore, we used an exogenous RNA immunoprecipitation (RIP) assay to verify the binding ability of HuR to the −150 to −21 motif at the ZO1 E23 splice site (Figure 5D and E).

GLTSCR1 decreases the transcription elongation rate of ZO1 to provide a time window for spliceosome recognition of the weak 3′ and 5′ splice sites in E23 and may also provide a time window for HuR to bind to the specific motif in ZO1 intron 22 to promote E23 inclusion. To verify this hypothesis, we used a mouse monoclonal anti-HuR antibody to evaluate the binding ability of endogenous HuR to the specific motif in ZO1 pre-mRNA in MOCK and GLTSCR1-KO cells. The RIP assay showed that GLTSCR1 KO decreased the binding ability of HuR to ZO1 pre-mRNA, but partial HuR binding ability was retained in GLTSCR1-KO cells (Figure 5F and G). This finding explained why knockdown of HuR in GLTSCR1-KO cells still inhibited E23 inclusion. To further demonstrate that HuR regulates E23 splicing by binding to ZO1 pre-mRNA, we constructed a ZO1 E23 minigene containing the full length of both intron 22 and intron 23 with a B-strength splice site (B) and a mutant minigene with deletion of the HuR-binding site in intron 22 (Mut B) (Supplementary Figure S6A). We then transfected these minigenes into HCT116 cells together with HuR siRNA. As shown in Figure 5H, E23 inclusion was decreased when HuR was knocked down by siRNA in the B group. Interestingly, deletion of the HuR-binding site resulted in the inhibition of E23 inclusion. However, HuR knockdown in the Mut B group did not affect E23 inclusion. These results demonstrated that HuR regulated E23 splicing depending on the motif in intron 22 of ZO1.

We validated that GLTSCR1 decreases the transcription elongation rate of ZO1 to provide a time window for HuR to recognize its binding motif in intron 22 of ZO1 and then promote E23 inclusion, a mechanism that corresponds to the kinetic model. Furthermore, the recruitment model of cotranscriptional regulation of AS proposes that Pol II can enrich abundant splicing factors in the vicinity of the pre-mRNA. To investigate whether GLTSCR1-mediated cotranscriptional regulation of ZO1 AS also fits the recruitment model, we used a coimmunoprecipitation (co-IP) assay to detect the interaction between Pol II and HuR in MOCK and GLTSCR1-KO cells. GLTSCR1 KO did not affect the interaction between Pol II and HuR (Supplementary Figure S6B). Consistent with this finding, HuR knockdown increased the invasion and migration of GLTSCR1-KO and MOCK cells (Figure 5I; Supplementary Figure S6C). These data demonstrated that HuR and GLTSCR1 mediated cotranscriptional regulation of ZO1 AS, as described by the kinetic model mediated, and promoted ZO1 E23 inclusion, which might play an antimetastatic role in CRC.

## Discussion

**Figure 6:**
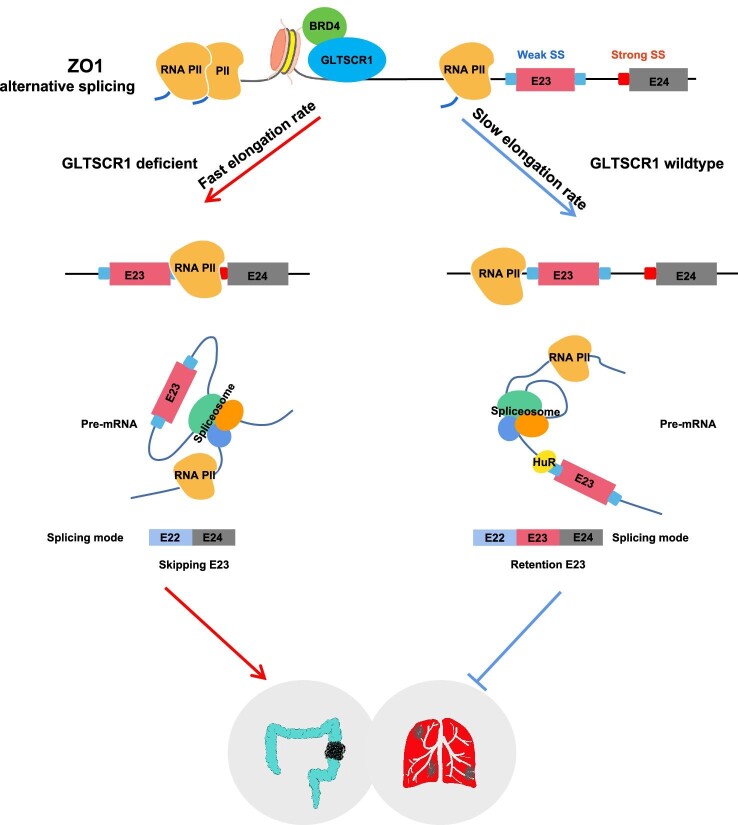
Model showing the regulatory role of GLTSCR1 in coupling transcription and AS. GLTSCR1 regulates ZO1 AS by inhibiting its transcription elongation, thereby inhibiting the progression of CRC.

As a key characteristic of cancer, aberrant AS can generate new cancer-specific markers and neoantigens, which have been considered important biomarkers for evaluating tumor progression and the therapeutic response (Climente-Gonzalez et al., 2017). Along with the development of new biotechnologies, the regulatory mechanisms of AS have been a constant research focus. Recently, AS has been shown to parallel transcriptional regulation in specific diseases, including cancer (Prochazka et al., 2014; Luo et al., 2017). Emerging evidence suggests that AS is controlled temporally and spatially by comprehensive regulation of the splicing, transcriptional, and chromatin organization machineries (Pal et al., 2012; Lev Maor et al., 2015). In this study, we identified GLTSCR1 as an AS regulator. Mechanistically, we reported a GLTSCR1-dependent transcription elongation regulation model for ZO1 E23 AS. Because of the weak 3′ and 5′ splice sites in E23, the ZO1 E23 AS event relies more heavily on the elongation rate. GLTSCR1 provides a low elongation rate condition for recognition of the weak splice sites in E23 by the specific splicing factor HuR, which promotes E23 inclusion. However, the E23+ variant of ZO1 showed a tumor-suppressive capability in CRC (Figure 6). This GLTSCR1-regulated AS model further elucidates the mechanism underlying the coupling of AS with transcription elongation. In addition, we evaluated AS and transcription elongation together with their biological functions to clarify a novel regulatory mechanism of CRC progression. Previous studies on AS in cancer biology have focused mainly on the *trans*-acting factors of cancer-associated genes, such as the well-characterized SR-rich proteins, hnRNP family members, and tissue-specific factors. The ability to study a specific genetic event without understanding its dynamic and spatial regulation is somewhat limited. Our data suggest a dynamic and spatial model for the regulation of AS by transcription elongation, allowing extensive study of AS regulation in CRC progression.

Currently, the SNPs rs1035938 and rs1052555 have been reported to be associated with the development and progression of oligodendroglioma (Yang et al., 2005). In addition, the expression of GLTSCR1 is associated with the progression of prostate cancer (Ma et al., 2018). Our previous study demonstrated that GLTSCR1 inhibited CRC metastasis by binding to BRD4 and blocking oncogenic transcription elongation. Specifically, a microsatellite instability frameshift mutation in exon 6 of GLTSCR1 produced two C-terminal-truncated proteins. However, these truncated GLTSCR1 proteins translocated into the cytoplasm and lost the BRD4-binding domain, which increased oncogenic transcription elongation. Based on our previous data, we assessed the regulatory roles played by the inhibition of GLTSCR1 transcription elongation in AS. The current results further supplemented the biological function of GLTSCR1 in CRC and provided a more systematic explanation of the molecular mechanism of tumor suppression. A recent report identified GLTSCR1 as a member of the noncanonical BRG-/BRM-associated factor (ncBAF) complex, which is required for transcription regulation and genome integrity (Alpsoy and Dykhuizen, 2018; Gatchalian et al., 2018; Inoue et al., 2019). However, the mechanism of GLTSCR1 as a subunit of ncBAF in cancer remains unknown; thus, the multidimensional regulatory roles of GLTSCR1 in tumorigenesis need further clarification.

ZO1 is the major component of the tight junction complex and interacts with occludins and claudins, which form barriers between adjacent cells to control the transport of water, ions, and macromolecules (Gonzalez-Mariscal et al., 2008). A previous study reported that RBPs such as SRSF6 and hnRNPL promote ZO1 E23 exclusion, while RBM47 induces ZO1 E23 inclusion (Heiner et al., 2010; Kim et al., 2019). Here, we proposed a novel GLTSCR1-dependent transcription elongation regulation model for ZO1 E23 AS based on the kinetic model of Pol II regulation in transcription, describing a more comprehensive regulatory mechanism of ZO1 AS. ZO1 E23 encodes an α-domain, and exclusion of ZO1 E23 results in remodeling of the F-actin structure. This alteration in F-actin might be closely related to the potential for tumor cell migration and invasion. However, the role of the α-domain and the functional differences between these two isoforms remain unclear. Although we demonstrated that GLTSCR1 played an inhibitory role in ZO1 E23 exclusion in CRC, no significant correlation of GLTSCR1 with ZO1 E23 AS was observed in CRC tissues because of the limited number of clinical samples. Thus, this finding requires further evidence.

In this study, we demonstrated a Pol II elongation rate-dependent ZO1 AS model in which GLTSCR1 reduced the elongation rate of ZO1, which provides a time window for recognition of the weak 3′ and 5′ splice sites in E23 by the spliceosome and the ZO1-specific splice factor HuR to promote ZO1 E23 inclusion. However, ZO1 E23 exclusion could promote CRC progression. Therefore, the splice sites in ZO1 E23 might be considered new therapeutic targets for CRC.

## Materials and methods

### Patient samples

Colorectal carcinoma and paired normal tissue samples (*n* = 64) from patients undergoing surgery in Wuxi Cancer Institute, the Affiliated Hospital of Jiangnan University, were included in this study. The research was approved by the Ethics Committee of Department of Medicine, Zhejiang University (2018-018), and all participating patients were informed. Tumor and paired normal tissue samples were prospectively collected from 2003 to 2011.

### Cell culture and treatment

Human CRC cell lines HCT116 and HCT8 were cultured in RPMI 1640 medium. Human HEK293T cell line was cultured in Dulbecco's modified Eagle's medium. All media were supplemented with glutamine, 10% fetal bovine serum (FBS), and penicillin/streptomycin. Cell lines were grown in a humidified atmosphere at 37°C with 5% CO_2_. HCT116 and HCT8 were purchased from the American Type Culture Collection (ATCC). HEK293T was purchased from the cell bank at the Chinese Academy of Sciences (Shanghai).

HCT116 MOCK and HCT116 GLTSCR1-KO cell lines were cultured as mentioned above. Cells were first treated with 300 µM DRB (Sigma, CAT#D1916) for 5 h and washed by phosphate-buffered saline (PBS) three times, and then fresh RPMI 1640 medium with 10% FBS and penicillin/streptomycin was added after DRB removal. Cells were harvested at 1-h intervals for RNA isolation and RT–qPCR. HCT116 MOCK and HCT116 GLTSCR1-KO2 cells were also treated with 50, 100, or 200 µM DRB for 3 h, washed by PBS three times, and harvested for RNA isolation and RT–qPCR.

Plasmids were transfected with LipoD293 (SignaGen). The siRNAs were transfected by using GenMute siRNA Transfection Reagent according to the instruction (SignaGen, CAT#SL100568).

### Transwell migration and invasion assay

Cell motility and invasion were measured by transwell and Matrigel chamber plates, respectively (24-well format; 8-μm pore size; Corning Costar). Briefly, 1 × 10^5^ cells were loaded per transwell cultured with serum-free media in the upside of the membrane. The cells were fixed in methanol for 10 min after migrating to the underside of the membrane and stained with crystal violet. Then, 30% glacial acetic acid was used to elute crystal violet to measure the cells on the lower face of the filter. Three independent experiments were performed in triplicate.

### Histological analysis

Mice colon and rectum samples were fixed with 4% formaldehyde and paraffin-embedded. The 4-µm-thick sections were stained with H&E.

### GLTSCR1^fl/^^fl^-Villin-Cre mouse generation and mouse colitis-associated CRC model

All animal experiments were performed in accordance with a protocol approved by the Institutional Animal Care and Use Committee at Zhejiang University (Ethics Committee number: 12169). GLTSCR1^fl/fl^ (C57) mice were bred with Villin-Cre mice to generate GLTSCR1^fl/fl^-Villin-Cre mice. AOM was injected intraperitoneally at 10 mg/kg body weight on Day 1 and followed by three cycles of DSS treatment. Each DSS-treated cycle contained 3 weeks. During the first week, mice were treated with DSS-containing water (2.0% *w*/*v*) and followed by two weeks of normal water. At the end of the three cycles, the mice were sacrificed (at the 11th week), and the colorectal tissues of the mice were dissected. After being cleaned by PBS, some of the tumor and normal tissues were stored in formalin for subsequent emplacement, while the others were taken for RNA extraction.

### RT–qPCR and transcription elongation assay

Total RNA from cells or tissues was isolated using the TRIzol reagent (Invitrogen). Then RT–qPCR was performed using Vazyme reagent (R223-01). After reverse transcription, qPCR analysis was performed using ChamQ™ Universal SYBR^®^ qPCR Master Mix (Vazyme, Q711-02/03). The data were analyzed using the ΔΔCT method and were first normalized to glyceraldehyde-3-phosphate dehydrogenase (GAPDH). The primers used for genomic qPCR are listed in Supplementary Table S1. RT–qPCR was used to measure the expression levels of ZO1 pre-mRNA and PICALM pre-mRNA.

### Immunofluorescence assay

Cells were washed with PBS, fixed with 4% formaldehyde for 10 min, and permeabilized with 0.1% Triton X-100 for 10 min. Then, the cells were blocked with 10% normal goat serum for 30 min, incubated with antibody overnight at 4°C, and incubated with specific anti-mouse or anti-rabbit secondary fluorescence antibody for 1 h. The slides were added with 200 μl of 100 nM rhodamine phalloidin and incubated at room temperature in the dark for 30 min. The cells were washed three times in PBS and then incubated with DAPI (Thermo Fisher) for 20 min.

### RNA-seq and AS event analysis

HCT116 MOCK and GLTSCR1-KO2 cells were collected and total RNA was extracted, sequenced, and analyzed by RiboBio. Three biological replicates were used for condition. The cDNA libraries were prepared from high-quality RNA using an Illumina TruSeq RNA Sample Prep Kit following the manufacturer's instructions (Illumina). The individual RNA-seq libraries were pooled based on their respective sample-specific 6-bp adaptors and sequenced at 150 bp/sequence pair read using an Illumina HiSeq 3000 sequencer. Gene differential expression and transcript differential expression analyses were accomplished by the Cuffdiff program in the Cufflinks package. Genes with two different isoforms were selected, which displayed opposite expression patterns. Gene isoforms with *P* < 0.05 and log2 fold change >0 and <0 were defined as differentially expressed isoform candidates. In addition, genes without significant change at gene level were selected for further analysis. All raw and processed sequencing data generated in this study have been submitted to the NCBI Sequence Read Archive (SRA, https://www.ncbi.nlm.nih.gov/sra) under accession number PRJNA517374.

### ChIP assay

HEK293T cells were cultured in a 10-cm culture dish. After 18 h, cells were transfected with Flag-GLTSCR1 by LipoD293 (SignaGen). Cells were prepared for ChIP assay by using anti-Flag antibody at 48 h after transfection. ChIP assays were performed by a SimpleChIP^®^ Enzymatic Chromatin IP Kit (Magnetic Beads) (Cell Signaling Technology, CAT#9003) according to the manufacturer's instructions. Samples were analyzed by real-time PCR using SYBR Green Power Master Mix following the manufacturer's protocol. ChIP–PCR analysis was performed to detect the accumulation of RNA Pol II in ZO1. The DRB treatment was described above.

### Brdu-labelled nascent RNA analysis

HCT116 MOCK and HCT116 GLTSCR1-KO2 cells were seeded in a 10-cm culture dish. After 18 h, cells were treated with bromouridine (Aldrich) at a final concentration of 2 mM for 2 h. The cells were washed with PBS three times, trypsinized, and collected. Total RNA from cells was isolated using the TRIzol reagent, and the BrdU-labelled nascent RNA was pulled down by anti-BrdU monoclonal antibodies (BD Biosciences, 555627) and then detected by RT–qPCR.

### RIP

Magna RIP Kit (Merck Millipore, CAT#17-700) was used, but the beads in the kit were replaced with M2 magnetic beads (Sigma-Aldrich), to pull down the proteins with Flag-tagged HuR. Cells were seeded in 10-cm plates and washed twice with 5 ml ice-cold PBS. Cell pellet was collected and resuspended in complete RIP lysis buffer. After using RIP wash buffer to wash 50 µl of M2 magnetic beads twice, 100 µl of lysis and beads were incubated in 900 µl of RIP immunoprecipitation buffer at 4°C overnight. Then, beads were washed by RIP wash buffer five times and collected by a magnetic separator. The immunoprecipitate was resuspended in 150 µl proteinase K buffer and incubated at 55°C for 30 min. The supernatant was transferred into 250 µl of RIP wash buffer and 400 µl of phenol:isoamyl alcohol in the tube. Then, the aqueous phase was moved into a new tube and mixed with 400 µl chloroform. Salt solution was added to enhance the precipitation of RNA at −80°C overnight. Finally, the pellet was washed by 80% ethanol and resuspended in 15 µl of RNase-free water.

### Quantifications and statistical analysis

Statistical specifications of each experiment precision measure and the statistical tests used are provided in figure legends. Kaplan–Meier survival analysis was performed using the software IBM SPSS Statistics 20 with the log-rank (Mantel–Cox) test.

## Supplementary Material

mjac009_Supplemental_File

## Notes

**Author contributions:** conceptualization by H.Z. and E.X.; methodology by F.H., B.Y., and M.Z.; investigation by F.H., B.Y., M.Z., Q.H., M.M., Z.H., and M.L.; resources by F.H., B.Y., M.Z., and M.M.; writing original draft by F.H., B.Y., and M.Z.; writing review and editing by H.Z. and E.X.; supervision by H.Z. and E.X.; and funding acquisition by H.Z.
