# Supplementary material for: GLTSCR1 coordinates alternative splicing and transcription elongation of ZO1 to regulate colorectal cancer progression
Source: J Mol Cell Biol. 2022 Feb 26;14(2):mjac009. doi: 10.1093/jmcb/mjac009 (PMC9188103; doi:10.1093/jmcb/mjac009)
Supplement: mjac009_Supplemental_File [file mjac009_supplemental_file.pdf]

Supplementary material

GLTSCR1 coordinates alternative splicing and transcription elongation of ZO1 to regulate colorectal cancer progression

Fengyan Han[1,†], Beibei Yang[1,†], Mingyue Zhou[2,†], Qiong Huang[1,3,4], Minglang Mai[1], Zhaohui Huang[2], Maode Lai[3,4,5], Enping Xu[1,3,4,*], and Honghe Zhang[1, 3,4,*]

[1] Department of Pathology and Women's Hospital, Zhejiang University School of Medicine, Research Unit of Intelligence Classification of Tumor Pathology and Precision Therapy, Chinese Academy of Medical Sciences (2019RU042), Hangzhou 310058, China

[2] Cancer Epigenetics Program, Wuxi School of Medicine, Jiangnan University, Wuxi, China

[3] Key Laboratory of Disease Proteomics of Zhejiang Province, Hangzhou 310058, China

[4] Cancer Center, Zhejiang University, Hangzhou 310058, China

[5] Department of Pharmacology, China Pharmaceutical University, Nanjing 210009, China

[†] These authors contributed equally to this work.

[*] Correspondence to: Enping Xu, E-mail: xep@zju.edu.cn; Honghe Zhang, E-mail: honghezhang@zju.edu.cn

Figure S1

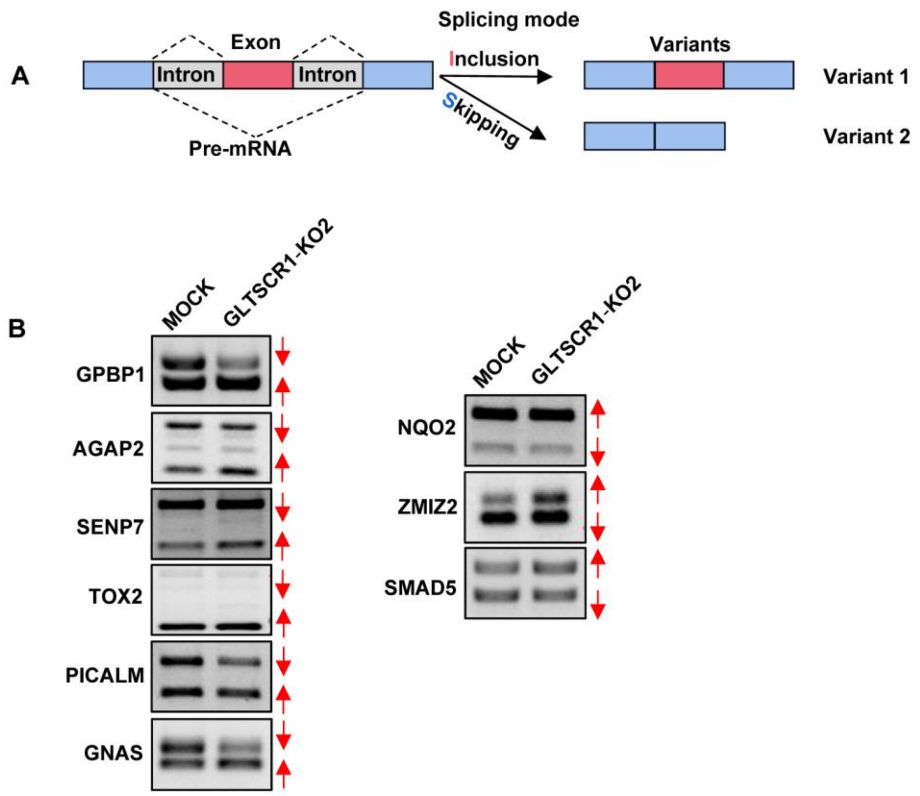

Supplementary Figure S1: GLTSCR1 regulates alternative splicing of genes.

A. Schematic diagram of Exon skipping.

B. RT-PCR analyses of representative GLTSCR1 affected cassette exons in original RNA-Seq HCT116 cells, as well as GLTSCR1-KO2 HCT116 cells.

Figure S2

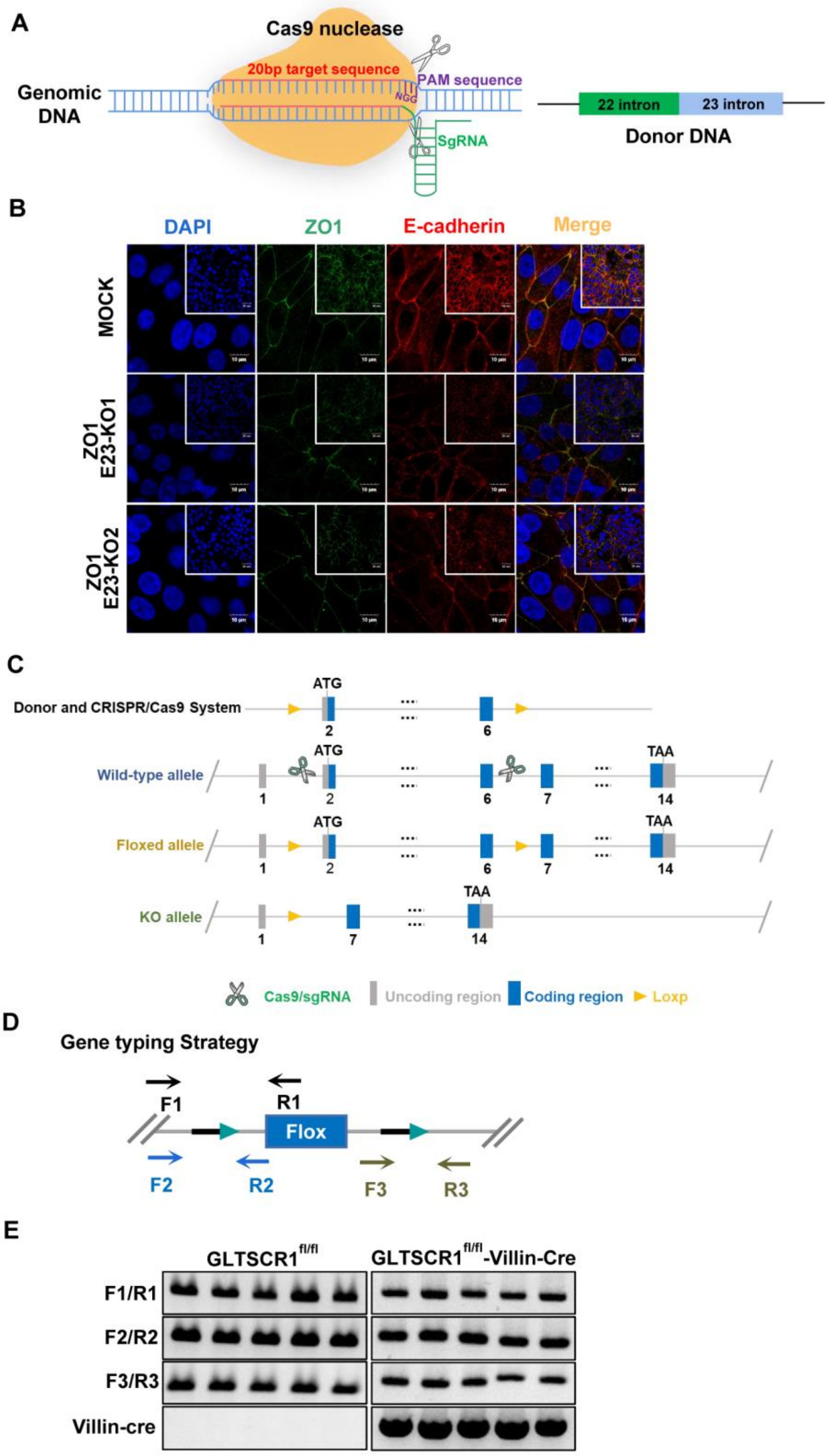

Supplementary Figure S2: ZO1 E23 inclusion isoform has a tumor suppression capability in CRC.

A. A model for knocking out exon 23 of ZO1 by CRISPR-Cas9.

B. Immunofluorescence showing position of ZO1 in MOCK and ZO1 E23 KO HCT8 cells. Cells were immunostained with anti-ZO1(green), anti-E-cadherin (red) and DAPI (blue). The scale bars of 150× is 10 μm and the scale bars of 60× is 30 μm.

C. Schematic overview of the GLTSCR1$^{fl/fl}$-Villin-Cre mice model.

D. Schematic overview of mice genotype detection strategy.

E. Identification of mice genotype by RT-PCR.

Figure S3

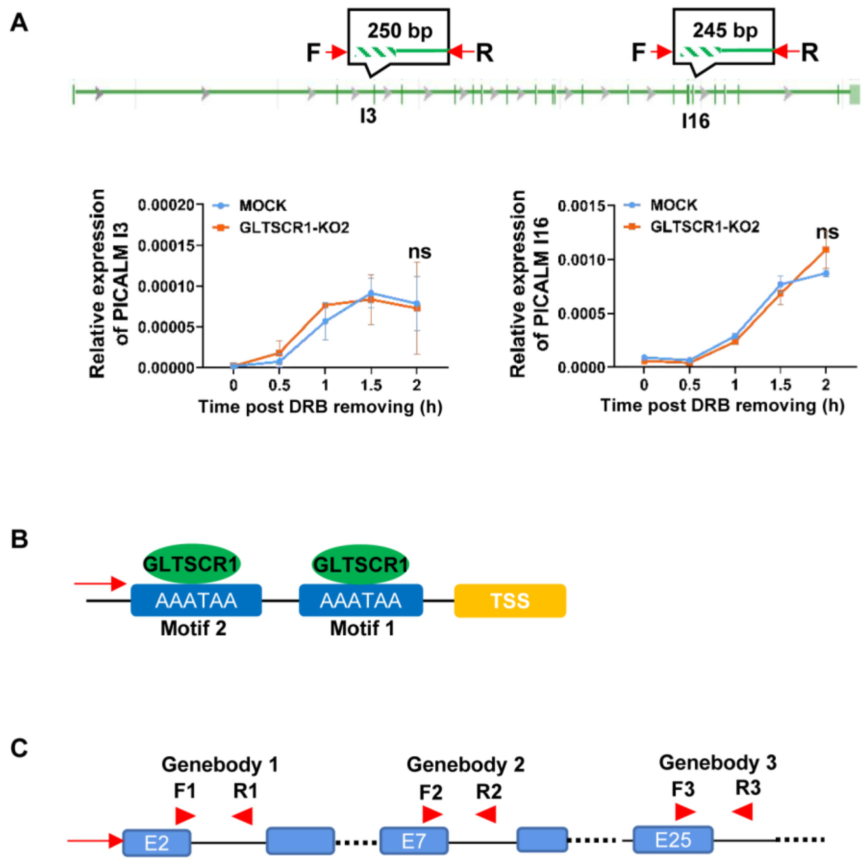

Supplementary Figure S3: GLTSCR1 regulates ZO1 E23 alternative splicing by inhibiting transcriptional elongation.

A. Transcription efficiency of PICALM in MOCK and GLTSCR1-KO2 HCT116 cells, as determined by RT-qPCR. The upper schematic diagram represents the primers sites of PICALM. Data are presented as mean ± SD; statistical significance was assessed by unpaired *t*-test. ns, not statistically significant.

B. Schematic overview of GLTSCR1 binding motif in the ZO1 promoter region.

C. Schematic diagram represents the primers sites of ZO1 gene for RNA Pol II ChIP-PCR.

Figure S4

A

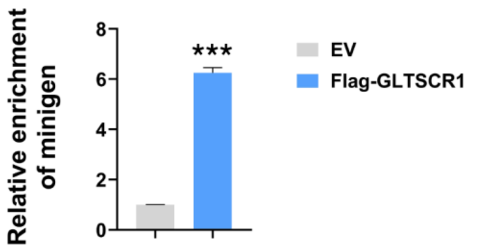

Supplementary Figure S4: GLTSCR1 bind to the promoter of minigene.

A. ChIP-PCR to detect GLTSCR1 binding ability to the promoter of minigene.

Figure S5

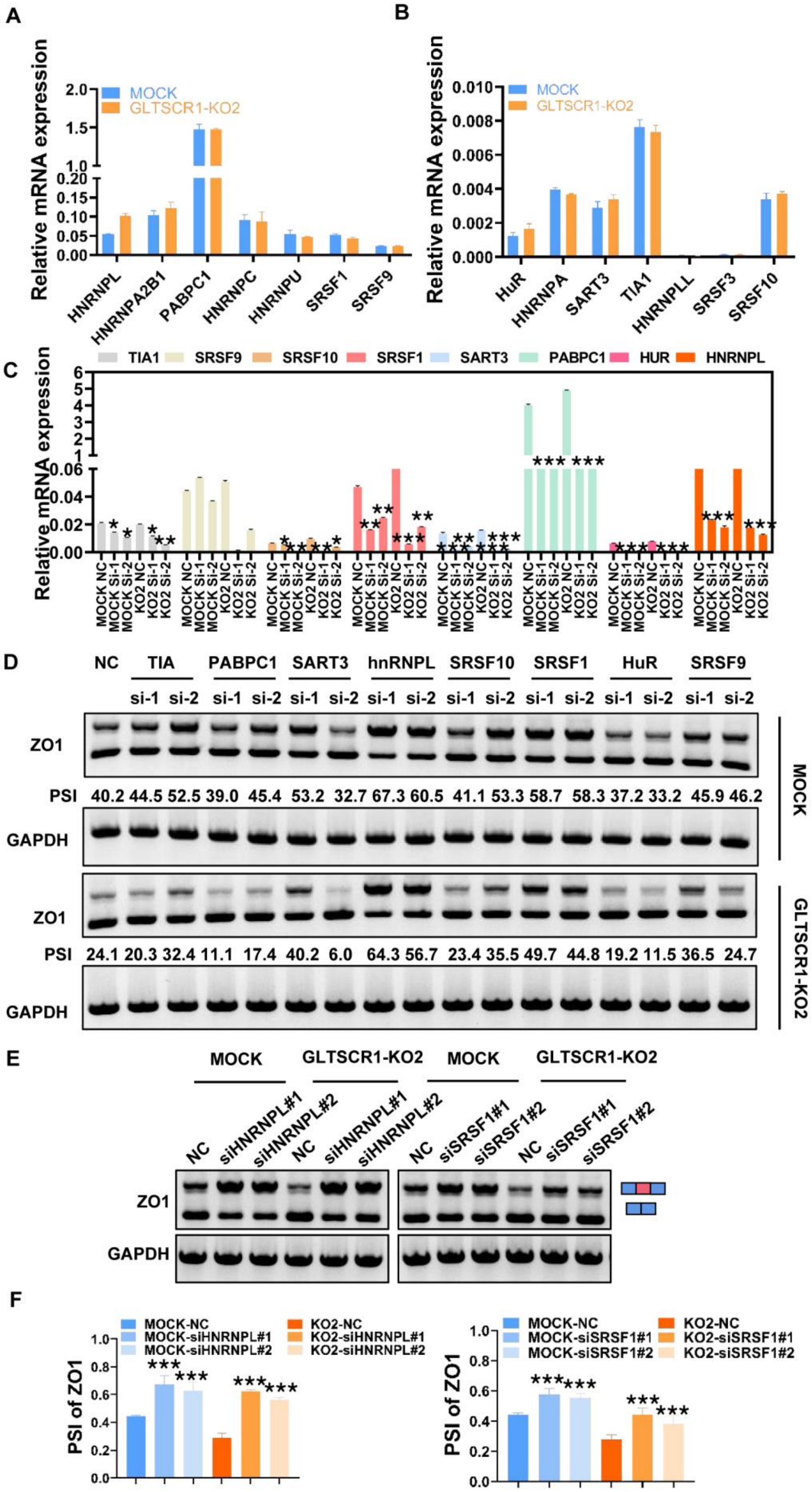

Supplementary Figure S5: HuR binds to ZO1 22nd intron and promotes ZO1 E23 inclusion.

A and B. RT-qPCR for detecting various splicing factors expression in MOCK and GLTSCR1-KO2 cells.

C. Knockdown of various splicing factors in MOCK and GLTSCR1-KO2 cells by respective siRNA. RT-qPCR was performed to detect the knockdown efficiency. Data are presented as mean ± SD; statistical significance was assessed by unpaired *t*-test. *$P<0.05$, **$P<0.01$, ***$P<0.001$; ns, not statistically significant; $n=3$.

D. RT-PCR for detecting ZO1 exon23 splicing isoforms expression; si-1 and si-2 represent specific siRNAs to different splicing factors.

E. siRNA results of hnRNPL and SRSF1.

F. The quantitative results of E.

Figure S6

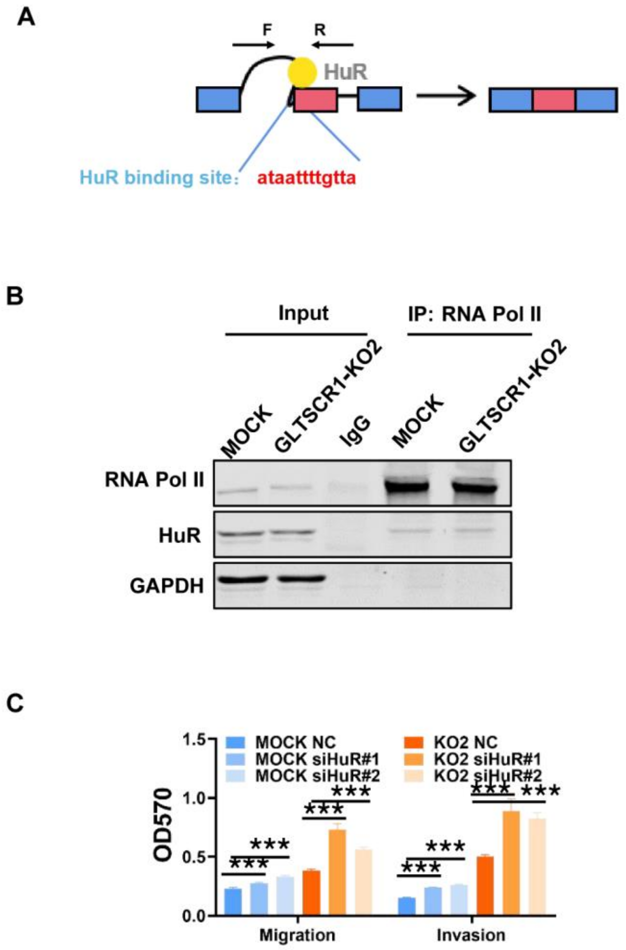

Supplementary Figure S6: HuR binds to ZO1 22nd intron and promotes ZO1 E23 inclusion.

A. Schematic diagram of ZO1 E23 alternative splice model and HuR binding motif.

B. CO-IP assay detect the interaction with HuR and Pol II in HCT116 MOCK and GLTSCR1-KO2 cells.

C. Histogram represents quantification analysis of transwell assay for investigating migration and invasion change by HuR knockdown by siRNA in HCT116 MOCK and GLTSCR1-KO2 cells. Data are presented as mean ± SD; statistical significance was assessed by unpaired *t*-test. *$P<0.05$, **$P<0.01$, ***$P<0.001$; ns, not statistically significant; $n=3$.

Supplementary Table S1: Primers used for genomic qPCR.

Gene	Forward	Reverse
GPBP1	TTTTCCGGCAGCATGGTAGT	TACCTCCATTAGGACGCCCA
AGAP2	GAACATCCACGGCAAGGAGA	TGCACACTCTGAACCCACAG
SENP7	AACGCTGGACTCTCCCTTTG	ATTGTGCGCAGTCTGCTTTG
PICALM	ATGACTGCACCAGCCATTGA	AGTTCATCAAAGCCCCCAGA
GNAS	GCTGGAGAATCTGGTAAAAGCA	CAATCGCCTCTTTCAGGTTGT
NQO2	AAAAGGTTCGGGAGGCTGAC	TGGCCACAGGAAGTATCGAG
ZMIZ2	AGCAGTATCTGCAAGGAGGC	GACATGTAAGGGACGCTGCT
SMAD5	GCGGCCGAGCTGCTAATAAA	CTTTCCAGCCCAACAATCGC
SRSF1	CAGGATTCATGGAGCGGGAT	CAAGGAGATTGCTCCAGCGT
SRSF3	ATGACTTCCTTGCAGGCTCT	CTGCCAACTGGGACTGTATGT
SRSF9	GAGGAATGGGCCTCCTACAA	GCATAACAGACATCCCCAGC
SRSF10	AGACTTGCGGCGTGAATTTG	AAATCCTCTTGGACGGCGAG
PABPC1	CTGTAATCAACCCCTACCAG	AGCACCGGGCATATTTTGGA
HuR	AAGCACCCGAAGACGGTTAG	GCTGCGAAAAGCACATGGAA
HNRNPA	GCAATAGCAGGTGGAACCCT	GGAGCCATTCGCGCTATACT
HNRNPA2B1	CGTGGATTTGGGGATGGCTA	TACCTCTGGGCTCTCATCCTC
HNRNPC	ACACTTCACGAAGGGGCAAA	TCATCCAGTAGGTCCCCCTC
HNRNPL	AACAATCGGTTCTCCACCCC	TTTCACAGAAGATGGCCGCT
HNRNPU	TGAGATTGCTGCCCGAAAGA	ACCGCATGTTCTGGTAGGTC
HNRNPLL	CATGGGTGGGCTTCAGAGAAT	GCTGTGAACTTGGTAACAATCG
SART3	AAGCTGCTCGGCTTGAGAAA	GACAAGGCAGTTCTCGACCA
TIA1	GGGTAGGGGCAAATGACTCC	TTGGTTGCATGCCCTGATCT
ZO1	GGAGTCTGCCATTACACGGT	AGGTCTCTGCTGGCTTGTTT
ZO1 I2	AGCTGGGCATGAAATGGTGA	GGCTCTCGTACTGGTGATGG
ZO1 E7	AGTTTGGCAGCAAGAGATGGC	ATATCCCTCTCCCCCAAACAC
ZO1 E25	GCGAGAAACGCTATGAACCC	ACTGTTCCCAGCCCAAAGGT
PICALM I3	GGCCAAAGACTACAATTCCCTTG	GGCTCGTTTTTGGTCACTTACG
PICALM I16	AACTACTGCTTCCACTGCCTATT	ATTAAGGTACTGCCATCTCACCA
ZO1 motif1	TAGTTACTGGATGGGGCATGG	AATCACAGGCATACACCACTA
ZO1 motif2	AGAGGTGCAGGAGGGATAAACT	CCATGCCCCATCCAGTAACTA
PIP ZO1	CCCAAAAAGTTTAGGACATGAGGT	GCGAAAGGTAAGGGACTGGA
PSE ZO1	GTCAAACAGCACCTTTGTGGT	TGCAGTAGTTCTCCAGTTGGT
siRNA		
PABPC1#1	GUAGGCAACAUAUUCAUUATT	UAAUGAAUAUGUUGCCUACTT
PABPC1#2	CACCUCACUAACCAGUAUATT	UAUACUGGUUAGUGAGGUGTT
TIA1#1	GGAGUUUCAUGAGCAUCGUTT	ACGAUGCUCAUGAAACUCCTT
TIA1#2	CUUGGUGGAAGACAAAUCATT	UGAUUUGUCUUCCACCAAGTT

HuR#1	GAGGCAAUUACCAGUUUCATT	UGAAACUGGUAAUUGCCUCTT
HuR#2	CCGUCACCAAUGUGAAAGUTT	ACUUUCACAUUGGUGACGGTT
HNRNPL#1	GCAGCCGACAACCAAAUAUTT	AUAUUUGGUUGUCGGCUGCTT
HNRNPL#2	GCACUCUGAAGAUCGAAUATT	UAUUCGAUCUUCAGAGUGCTT
SART3#1	GCCAGAAGAUGAGUGAAAUTT	AUUUCACUCAUCUUCUGGCTT
SART3#2	GGCCACAUUUGCAGAGUAUTT	AUACUCUGCAAAUGUGGCCTT
Negative Control	UUCUCCGAACGUGUCACGUTT	ACGUGACACGUUCGGAGAATT
